# Somatosensory Cortices Are Required for the Acquisition of Morphine-Induced Conditioned Place Preference

**DOI:** 10.1371/journal.pone.0007742

**Published:** 2009-11-03

**Authors:** Zhiqiang Meng, Chang Liu, Xintian Hu, Yuanye Ma

**Affiliations:** 1 Laboratory of Sensory-motor Integration Research and State Key Laboratory of Brain and Cognitive Science, Kunming Institute of Zoology, the Chinese Academy of Sciences, Kunming, Yunnan, People's Republic of China; 2 Laboratory of Primate Cognitive Neuroscience and State Key Laboratory of Brain and Cognitive Science, Kunming Institute of Zoology, the Chinese Academy of Sciences, Kunming, Yunnan, People's Republic of China; 3 Graduate University of the Chinese Academy of Sciences, Beijing, People's Republic of China; 4 Kunming Primate Recearch Center, the Chinese Academy of Sciences, Kunming, Yunnan, People's Republic of China; 5 Kunming Biomed International, Kunming, Yunnan, People's Republic of China; Hotchkiss Brain Institute, University of Calgary, Canada

## Abstract

**Background:**

Sensory system information is thought to play an important role in drug addiction related responses. However, how somatic sensory information participates in the drug related behaviors is still unclear. Many studies demonstrated that drug addiction represents a pathological usurpation of neural mechanisms of learning and memory that normally relate to the pursuit of rewards. Thus, elucidate the role of somatic sensory in drug related learning and memory is of particular importance to understand the neurobiological mechanisms of drug addiction.

**Principal Findings:**

In the present study, we investigated the role of somatosensory system in reward-related associative learning using the conditioned place preference model. Lesions were made in somatosensory cortices either before or after conditioning training. We found that lesion of somatosensory cortices before, rather than after morphine conditioning impaired the acquisition of place preference.

**Conclusion:**

These results demonstrate that somatosensory cortices are necessary for the acquisition but not retention of morphine induced place preference.

## Introduction

Somatosensory cortices are involved in various forms of sensory processing such as pain, touch et al. It has been reported that sensory cortices are highly plastic to reflect recent experience and learning in adult animals [1]–[4]. The capacity of plasticity in cortical areas is one of the most salient features to explain development, learning, or recovery of function [5].

Sensory system information plays an important role in drug addiction related responses [6]. For example, administration of cocaine or the presentation of drug-related cue can enhance evoked responses in the primary sensory cortex of experimental animals and humans [7], [8]. In addition, changes of activity in the somatosensory cortices are associated with the euphoric experience of acute effects of opiate and stimulant drugs [9], [10]. Moreover, a recent study demonstrated that the hypocretin transmission plays a significant part in rewarding properties of nicotine, and those hypocretin neurons and hypocretin-1 receptors innervate the somatosensory cortex [11]. Based on these findings, it seems that somatosensory cortices are possibly and potentially in operation of drug addiction. However, how sensory cortices participate in drug addiction is still unclear.

Recent theories of drug addiction emphasized learning processes [12], [13], and the work in animals suggested that learning and memory could be affected by the circuits within which drugs of abuse act [14]. It is now widely accepted that addiction is a memory of the state of body and cue, and is first acquired through processing in the cortex where synthesizes visual, auditory and somatic information. However, little is known about the role of the somatosensory system in this drug-related associative learning behavior, although the interoceptive system is found monitors bodily changes at initial stages of drug using [15], [16].

In the present study, we investigated the role of somatosensory cortex in drug -related learning and memory using the morphine conditioned place preference (CPP) paradigm which measures a learning process and provides unique information about the rewarding effect of contextual cues associated with a drug stimulus [17], [18]. CPP involves not only the formation session (conditioning), but also the retrieval session (preference test) of drug-associated memories [12].To elucidate of the participation of sensory cortices in reward-related associative learning, we produced lesion of bilateral somatosensory cortices by electrolytic electrodes either before or after conditioned training. Our results reveal that somatosensory cortices are necessary for the acquisition of conditioned place preference in rats.

## Materials and Methods

### Ethics statement

All experiments were conducted during light phase, and in accordance with the procedures approved by Animal Experimental Committee, Kunming Institute of Zoology, Chinese Academy of Sciences, and with the National Institutes of Health Guidelines for the Care and Use of Laboratory Animals (NIH Guidelines).

### Subjects

Adult male Sprague-Dawley rats (250–300 g, from Kunming Medical University, Kunming, China) were housed in a temperature-controlled room (23±1°C) in a 12 hr light/dark cycle (light on 8:00 a.m. to 8:00 p.m.). Food and water were available ad libitum.

### Electrode implantation

Forty four rats were randomly assigned to five groups: eight rats for saline group, morphine group and sham group; ten rats for two sensorycortex lesion groups. Only the sham and lesion groups underwent the surgery. Rats were first treated with atropine in order to reduce mucous secretion and then sodium pentobarbital anesthesia (60 mg/kg i.p.). Body temperature was maintained at normothermia using a heating pad. When rats were in a deep level of anesthesia as indicated by a slow respiratory rate and lack of response to tail pinch, they were placed into a stereotaxic apparatus. Previously prepared concentric bipolar electrodes were implanted into both left and right brain hemisphere aiming at the whole secondary somatosensory cortex (S2) and the adjacent primary somatosensory cortex (S1). S2 is important for multisensory integration besides its function in unisensory processing [19]. Somatoensory information processing between S1 and S2 may be serial and/or parallel [20], [21]. So we made lesion of S2 and adjacent S1 areas. The outer electrode was Epoxy coated except the 0.5 mm tip, and the inner electrode of the inner brain side was 1 mm longer than the outer electrode and with the Teflon coated except 0.5 mm tip. The overall outside diameter was 0.5 mm. The tips were placed at the following coordinates with respect to bregma: 1.0 mm posterior, 6.5 mm lateral, 6.5 mm ventral. Dental cement was used to fix the electrodes to the skull. Two short exposed wirings from outer and inner electrode respectively were uncovered for latter lesion process. After surgery, the animals were injected with a dose of 100,000 U of benzyl-penicillin intramuscular as antimicrobial prophylaxis, and were allowed a postoperative recovery for 1 week before the experimental protocol.

### Lesion

Lesions were made by passing a direct current of 0.4 mA for 60 s through the electrodes. There were two lesion groups: one group of rats were undergone lesions just after surgery (L-CPP group), while the other group of rats were undergone lesions after the last conditioning session (CPP-L group). Animals in the sham group were treated with the same manipulation with CPP-L group except without current passing.

### Apparatus

A place preference apparatus consisted of two distinct xylary conditioning environments and a separated interim chamber with two guillotine doors. Each conditioning environment measured 45×45×30 cm. One environment was striped horizontally in an alternation of 5 cm black and white painting on the walls, while the other environment was striped vertically in the same pattern. The floor of the apparatus was textured on the horizontal side, but smooth on the vertical side. The interim area measured 45×22.5×30 cm, and was painted white with a very smooth floor. The activity of each subject was recorded by a video camera mounted above the center of the CPP apparatus. The time spent in each compartments was counted offline. The position of a rat was defined by the position of its body (forelimbs and head).

### Behavioral procedure

The conditioning protocol was divided into three periods: preconditioning (1 day), conditioning (8 days), post conditioning (1 day).

During pre-conditioning phase, animals were placed in the center of the interim chamber with two guillotine doors opened. They were allowed to explore the entire apparatus for 15 min for adaptation to this new environment. The time they spent in each chamber was recorded as pre-conditioning preference baseline. All subjects were given counter-balanced assignments so that half were conditioned with morphine in the vertically striped side and half in the horizontally striped side.

During the conditioning phase, rats were confined to either morphine side 5 min after morphine injection (10 mg/kg, dissolved to 1 ml with saline) or saline side after physiological saline injection (1 ml) for 50 minutes each trial. Four morphine pairing trials and four saline pairing trials were conducted on eight alternate days. The combination of the injections (morphine or saline) and the two compartments was counterbalanced across subjects [22]. The post conditioning test phase was carried out 24 hours after the last conditioning. Rats were placed in the interim chamber of the apparatus with the doors opened and allowed free access to the conditioning compartments for 15 min. The time they spent in each compartment was recorded during this 900 s drug-free test session and used as the preference score.

### Histology

At the end of behavioral testing, rats were deep anesthetized with sodium pentobarbital (60 mg/kg, i.p.). Rats were then transcardially perfused with 0.9% saline followed by 10% formalin. When the brains were removed out, they were post-fixed in 10% formalin. After fixation, brains were sliced into 30–40 um coronal sections and every 3^rd^ slice was mounted onto gelatin-coated slides which were stained using standard HE staining. Light and digitized images were evaluated for measuring the location and the extent of lesion with reference to a brain atlas [23].

### Statistical analysis

CPP was demonstrated by the time spent in the morphine-paired and vehicle-paired compartments. Only the rats with more than 2 entries to the two compartments during the post-conditioning test session were included in analyses. All behavioral data were presented as mean±S.E.M. A two-way repeated ANOVA was used to compare the time spent in the morphine paired compartment, post- vs. pre- conditioning tests as within subject factor and different treatments as between subject factor. Two-way ANOVA was used to analyze the numbers of entries into the two compartments for each rats. The percentages of time spent in the morphine paired side of each group were analyzed as following: 1. one-sample t test was used to test the effect of morphine treatment on CPP (morphine treatment groups vs. saline group); 2. Independent sample t test was used to test the surgery effect on morphine induced CPP (sham group vs. morphine group). 3. One-way ANOVA was used to test the lesion effects on morphine induced CPP. A P-value of 0.05 was set as the level of statistical significance for all statistical analyses. All statistical procedures were performed using SPSS software.

## Results

### Histology verification

**Figure 1:**
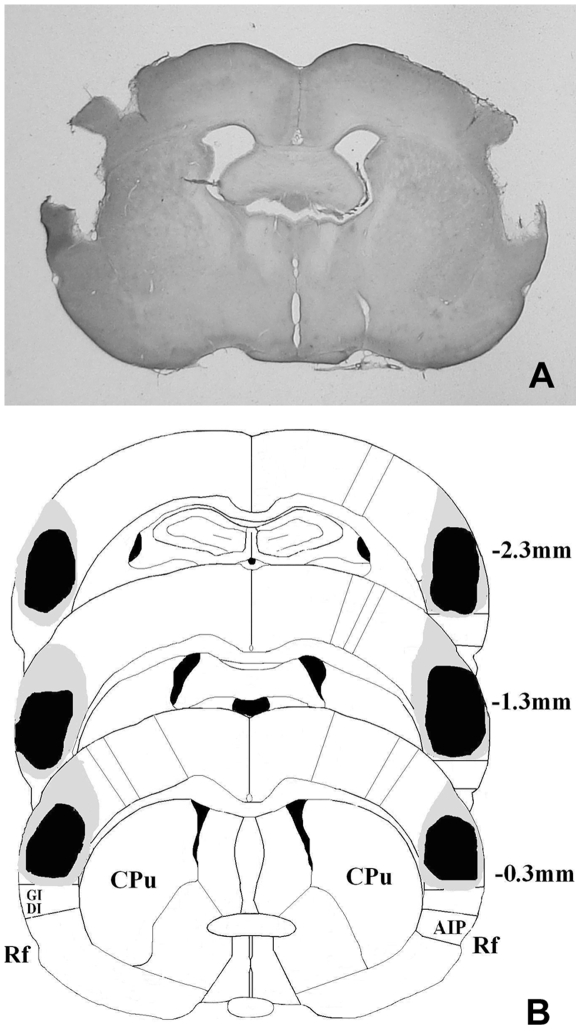
Histological localization of lesion sites. (a) A representative photomicrograph of H.E stained coronal sections shows a typical electrolytic lesion of S2 and adjacent S1. (b) A schematic representation of the anatomical location of damaged regions by an electrolytic lesion on the coronal section adapted from the atlas of Paxinos and Watson. The black area and grey area mark the smallest and the largest lesion respectively. Numbers indicate anterior-posterior (AP) distances (in mm) from bregma. CPu, caudate putamen; AIP, agranular insular cortex, posterior; DI, dysgranular insular cortex; GI, granular insular cortex; Rf, rhinal fissure.

Histological verification of lesion location was performed after behavioral testing. Lesion rats were included in the analyses if they met the criteria: more than 90% of the S2 and adjacent S1 were damaged and no or slight damage to adjacent areas. The assessment of damage to the target brain regions is presented in Fig. 1. Histological examination of the lesions showed that 6 rats from L-CPP group and 7 rats from CPP-L group adequately met the criteria to be included in the analyses.

### Behavioral results

**Figure 2:**
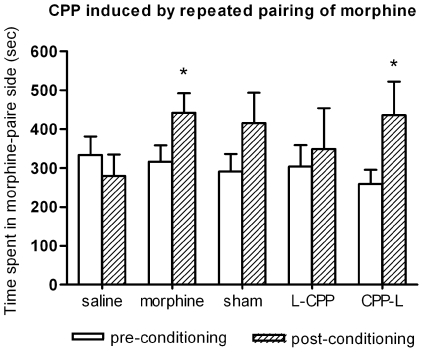
Place preference induced by morphine conditioning. The preference was determined by a comparison between post-conditioning test and pre-conditioning baseline. Total time (seconds) spent in morphine paired compartment was expressed as mean±S.E.M. Numbers of animals: saline, n = 7; morphine, n = 6; sham, n = 8; L-CPP, n = 6; CPP-L, n = 7. * p<0.05.

**Figure 3:**
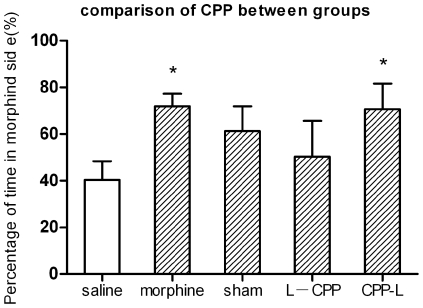
Comparison of place preference induced by different treatments. Data expressed as the proportion of time spent in the morphine paired compartment to a total time spent in both morphine and saline compartments. All comparisons were compared with saline control group. Numbers of animals: saline, n = 7; morphine, n = 6; sham, n = 8; L-CPP, n = 6; CPP-L, n = 7. * p<0.05.

**Figure 4:**
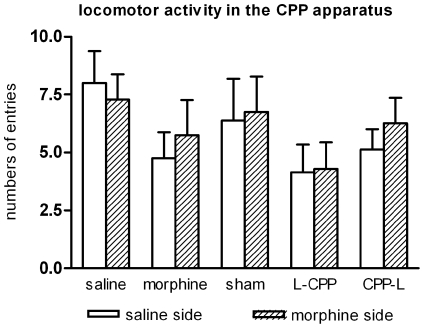
Numbers of entries to each compartment during the post-conditioning test. Data showed numbers of entries to the two conditioning compartment of each group. Data are expressed as mean±S.E.M. Student's t test revealed no significant difference between two compartments in any group. Numbers of animals: saline, n = 7; morphine, n = 6; sham, n = 8; L-CPP, n = 6; CPP-L, n = 7.

Morphine-induced place preference was presented in Fig. 2. Two-way repeated measures showed significant difference between post- and pre-conditioning tests (F (1, 68) = 6.552, p<0.05). Compared with the pre-conditioning test session, a profound place preference was produced by repeated pairings of morphine and the paired environment in morphine group (F (1, 10) = 11.479, p<0.05) and CPP-L group (F (1, 14) = 8.03, p<0.05). No significant place preference was found in the saline group, the sham group and the L-CPP group (all p>0.05). However, the sham group showed an obvious preference for the morphine side (F (1, 14) = 3.363, p = 0.1). Fig. 3 showed the CPP induced by each treatment. The results were represented as the proportion of time spent in the morphine paired compartment to total time spent in both morphine and saline compartments. One-sample t test showed that, compared with saline group, there is significant place preference for morphine control group (t (5) = 5.78, p<0.05) and CPP-L group (t (7) = 3.17, p<0.05). No preference was found for the L-CPP group (t (5) = 0.64, p>0.05) and sham group (t (7) = 1.95, p = 0.09). Independent sample t test showed that surgery itself can't impair conditioned place preference (sham vs. morphine control: t (12) = 0.801, p>0.05). One-way ANOVA showed that there is no significant difference between lesion groups and sham group (F (2, 19) = 0.746, p>0.05). When compared the numbers of entries to each compartment during the post-conditioning test. there was no significant difference between the two conditioning sides (F (1, 68) = 0.213, p>0.05). Each subject entered almost the same times into morphine side as saline side (Fig. 4).

## Discussion

In this study, we investigated the role of somatosensory cortices in reward-related learning and memory. We made lesions of bilateral S2 and adjacent S1 using electrolytic electrodes both before and after morphine conditioning. We found that lesions of somatosensory areas before rather than after morphine conditioning abolished the acquisition of the association between morphine and the specific environment, which was expressed as a conditioned place preference.

Somatosensory cortices participate in morphine-induced associative memory formation via two possible mechanisms. One is that somatosensory cortex might act as a modulator that strengthen the context-drug association. Neuroanatomy studies showed that somatosensory cortex is interconnected with the hippocampus, which underlies the learning of association with environmental context and drug effect [24]. In addition, somatosensory cortex also interacts with various brain networks such as the prefrontal cortex [25], the amygdala [26] and the dorsal striatum [27]. Several of these regions are involved in the acquisition and reinstatement of drug addiction related behaviors [28], [29]. Although how the information process within these brain regions is unclear, it is possible for the somatosensory cortices to facilitate CS-US (CS, conditioned stimulus; US, unconditioned stimulus) associations. This kind of function has been found in nucleus accumbens shell which is a key part of rewarding circuits [30], [31].

Another possible mechanism is that somatic sensory information, which is mainly encoded in sensory cortex, directly mediates the rewarding effect of drugs of abuse. It is still ambiguous what becomes associated with the context CS during conditioning. A widely accepted answer is that the stimulus conditions are produced by the drug [32]. Here, our results suggested that somatic sensory information might be a critical part of the nature of rewarding effect of drugs. This is consistent with a previous CPP study which demonstrated that dopamine D3 receptor in the somatosensory cortex participated in the morphine effects [33]. In addition, our previous results showed that multiple neurochemical changes in somatosensory cortices were induced during morphine administration [34]. Thus, somatosensory cortices might be part of the neural substance which mediating the rewarding effect of drugs. Furthermore, the rewarding effects of addictive drugs are multidimensional [35]. many sensory modalities contribute to this process such as gustatory, olfactory, visual element and somatosensory.

Dopamine has been widely implicated as the central mechanism through which drugs of abuse produce their effects [31]. Rewarding properties of morphine are produced via inhibition of GABAergic midbrain interneurons that negatively regulate dopamine neuron firing and dopamine release [36]. In the conditioned place preference paradigm, the memory of the association between sensory pleasure and specific environment is crucial for the acquisition and retention of the conditioned preference. This process is mediated by not only the mesolimbic and mesocorical dopamine projections, but also by their widely distributed network interactions with somatosensory cortex, amygdala and hippocampus [9]. Furthermore, discrete neurocircuits have been revealed that mediate different stages of the addiction cycle [37]. Thus, our results may suggest that somatosensory cortices are involved in the acquisition but not other stages.

Our present results showed that the somatosensory cortices are crucial for the acquirement of the association of a positive affective state with a specific environmental context. However, somatosensory cortices are neither required for the retention nor for the retrieval of drug associated memory which is in accordance with previous studies [11], [16]. They demonstrated that the inactivation of somatosensory cortex after conditioning has no effect on drug seeking behaviors. These results may suggest that somatosensory cortices are necessary element for the acquisition of drug associative memory. It is still unclear where these memories are stored and how to retrieval them. At least, our present results indicate that these sensory cortices are not necessary for the retention or retrieval of drug related memory. In addition, the interoceptive system, but not the somatosensory system is critical for negative feeling of the withdrawal from drugs, thus it mediates the urge of drug seeking [38]. Therefore, the somatosensory system and the interoceptive system plays different roles in addiction processes.

In the present study, we investigated the role of somatosensory cortex in CPP using electrolytic lesions technique. It is well known that electrolytic lesions often damage both the neuronal structure and axons passing through the area. Although our results demonstrated that somatosensory cortices are necessary for the acquisition of CPP, we can't specify whether somatosensory cortex or fibers passing through it to other areas were involved in this process. This should be clarified in future study via more specific treatments. Our result showed that the preference of the sham group was also abolished, and there was no significant difference between the lesion group and the sham group despite that a clear trend was found (see Fig. 2 and Fig. 3). A possible reason is that the implanted electrodes caused some mild damages to the somatosensory cortex, although no current passed through them.

Our present results demonstrated that the somatosensory system is required for the positive rewarding property of drugs. And previous studies indicated that interoceptive system is critical for the negative feeling of the withdrawal from drugs. Taken together, separate neural systems may subserve the positive rewarding effect of drugs and the negative feeling of withdrawal [39]. In conclusion, our results help a further understanding of the mechanisms of drug addiction, which depend upon their positive reinforcing and hedonic effects, and an avoidance of the negative, aversive consequences of withdrawal [40].
